# The Effect of Repetitive Transcranial Magnetic Stimulation on Dysphagia After Stroke: A Systematic Review and Meta-Analysis

**DOI:** 10.3389/fnins.2021.769848

**Published:** 2021-11-15

**Authors:** Weiwei Yang, Xiongbin Cao, Xiaoyun Zhang, Xuebing Wang, Xiaowen Li, Yaping Huai

**Affiliations:** ^1^Rehabilitation Department, Shenzhen Longhua District Central Hospital, Shenzhen, China; ^2^Neurology Department, Shenzhen Longhua District Central Hospital, Shenzhen, China

**Keywords:** deglutition disorders, transcranial magnetic stimulation, stroke, meta-analysis, systematic review

## Abstract

**Objective:** The primary purpose of our study is to systemically evaluate the effect of repetitive transcranial magnetic stimulation (rTMS) on recovery of dysphagia after stroke.

**Search Methods:** We searched randomized controlled trials (RCTs) and non-RCTs published by PubMed, the Cochrane Library, ScienceDirect, MEDLINE, and Web of Science from inception until April 24, 2021. Language is limited to English. After screening and extracting the data, and evaluating the quality of the selected literature, we carried out the meta-analysis with software RevMan 5.3 and summarized available evidence from non-RCTs.

**Results:** Among 205 potentially relevant articles, 189 participants (from 10 RCTs) were recruited in the meta-analysis, and six non-RCTs were qualitatively described. The random-effects model analysis revealed a pooled effect size of SMD = 0.65 (95% CI = 0.04–1.26, *p* = 0.04), which indicated that rTMS therapy has a better effect than conventional therapy. However, the subgroup analysis showed that there was no significant difference between low-frequency and high-frequency groups. Even more surprisingly, there were no statistically significant differences between the two groups and the conventional training group in the subgroup analysis, but the combined effect was positive.

**Conclusion:** Our study suggests that rTMS might be effective in treating patients with dysphagia after stroke.

## Introduction

Dysphagia is one of the common complications after stroke, with a high incidence of 37–78% (Cola et al., 2010). Dysphagia can cause malnutrition, pneumonia, dehydration, etc., which will significantly increase the death rate of patients. Edmiaston et al. (2010) found that the mortality rate of patients with dysphagia after stroke was three times as high as stroke patients with normal deglutitive function. Therefore, rehabilitation training for dysphagia after stroke is still an urgent problem to be solved in clinical practice.

At present, repetitive transcranial magnetic stimulation (rTMS) is a non-invasive brain stimulation method for use in treating post-stroke dysphagia. In general, rTMS can be divided into two primary treatment regimes: low-frequency rTMS (<1 Hz) and high-frequency rTMS (≥1 Hz) according to its stimulation frequencies. Low-frequency rTMS (LF-rTMS) inhibits cortical excitability, while high-frequency rTMS (HF-rTMS) activates cortical excitability. It can modulate human cortical and subcortical structures both at the site of stimulation and in remote areas (Bestmann et al., 2004; Valero-Cabré et al., 2017). The repeated electrical stimulation could produce a cumulative effect, which causes nerve cells to produce action potential, promote the release of neurotransmitters and regulation of synaptic plasticity to improve neurological outcome (Michou et al., 2014). Moreover, rTMS can modify excitability of the cerebral cortex at the stimulated site and also at remote areas along functional anatomical connections (Kobayashi and Pascual-Leone, 2003). Up to now, it has been recognized that rTMS can inhibit maladaptive cortical plasticity and improve adaptive cortical activity to promote neurological rehabilitation in stroke patients. According to the latest TMS guidelines (Rossini et al., 2015), rTMS has demonstrated level “A” efficacy in the rehabilitation of depression and neuropathic pain.

As a non-pharmacological strategy, rTMS could explore cortical circuits by the measurement of motor evoked potential and cortical silent period. On the other hand, it can search the related neurochemical pathways in neurological disorders and induce cortical plasticity to achieve the purpose of treatment (Bordet et al., 2017). Some experiments and reviews have shown that the mechanism of action of rTMS mainly depends on the long-term depression (LTD) and long-term potentiation (LTP) (Ziemann, 2004; Lanza and Ferri, 2019). Additionally, the change of synaptic plasticity and neurotransmitter release are also the way for therapeutic role (Ziemann, 2004; Lanza and Ferri, 2019).

In recent years, increasing number of clinical studies and reviews have shown that rTMS can be new diagnostic and therapeutic tools for assessment and rehabilitation in motor and cognitive impairments (Fisicaro et al., 2019; Di Lazzaro et al., 2021). However, since the nerve conduction pathways of dysphagia are complex, the effects of rTMS on the improvement of dysphagia after stroke varies according to the stimulation on site, frequency, time, and other parameters. Currently, there are no detailed uniform standards for the clinical practice of rTMS on post-stroke dysphagia. Therefore, we conducted qualitative systematic review and quantitative meta-analysis of relevant clinical trials, aiming to provide objective evidence-based medical evidence for the effect of rTMS on post-stroke dysphagia.

## Materials and Methods

### Search Strategy

We systematically searched five databases including PubMed, Cochrane Library, ScienceDirect, MEDLINE, and Web of Science for relevant studies, which were published through April 24, 2021. The retrieval language was limited to English, and the search strategy was designed by the combination of MeSH words and free words. MeSH words include deglutition disorders, stroke, and transcranial magnetic stimulation, and free words include swallowing disorders, dysphagia, cerebrovascular accident, apoplexy, cerebrovascular apoplexy, repetitive transcranial magnetic stimulation, and rTMS.

### Inclusion and Exclusion Criteria

Two reviewers (Weiwei Yang and Xiaoyun Zhang) independently screened the titles and abstracts by applying the same inclusion and exclusion criteria. Any disagreements were resolved by consensus with the third (Yaping Huai).

#### Inclusion Criteria

The inclusion criteria of this meta-analysis were as follows: (1) all patients with ischemic or hemorrhagic stroke displayed definitive radiographic evidence of relevant pathology on magnetic resonance imaging (MRI) or computed tomography (CT); (2) dysphagia symptoms were investigated in all patients; (3) no participants had other neurological diseases or other swallowing disorders; and (4) the experimental group received rTMS training, and the control group received routine rehabilitation training or other rehabilitation training.

#### Exclusion Criteria

Exclusion criteria were as follows: (1) patients with unstable tachyarrhythmia, fever, infection, seizures, and sedative use; (2) patients had severe cognitive impairment or aphasia; (3) non-English publications; (4) papers that do not provide data of interest; (5) reduplicated articles; and (6) reviews, abstracts, case reports, and non-clinical studies.

### Data Extraction and Quality Assessment

After screening the titles and abstracts, two evaluators (Weiwei Yang and Xiaoyun Zhang) independently assessed eligibility for inclusion in the analysis and extracted the relevant material according to predefined inclusion and exclusion criteria. We retrieved full-text articles for references for which a decision on eligibility could not be made based on title and abstract alone. Another two researchers (Xiongbin Cao and Xuebing Wang) then performed quality evaluation of the selected articles according to the Cochrane Handbook for Systematic Reviews of Interventions 5.1.0. The criteria consist of seven parts: random sequence generation, allocation concealment, blinding of participants and personnel, blinding of outcome assessment, incomplete outcome data, selective reporting, and other sources of bias. If the above quality standards are fully met, which indicates that the overall risk of bias is low and the study quality is high and the studies are rated as Grade A, while one or more of the standards are met, the risk of bias is moderate and the studies are defined as Grade B, and if the above standards are not met, the risk of bias is high and the quality of the studies is rated as Grade C. In cases of disagreement, a third person (Xiaowen Li) was consulted to reach a consensus.

### Outcome Indicators

For the included RCTs, we calculated the mean scores (Mean) and standards deviations (SDs) before and after interventions according to guidance in the Cochrane Handbook for Systematic Reviews of Intervention. Outcome measures for the efficacy of therapy were as follows: (1) PAS (Penetration–Aspiration Scale) and (2) DD (Dysphagia Grade). The PAS is an eight-point scale that measures selected aspects such as penetration and inhalation, depth of invasion into the delivery airway, and whether substances entering the airway are expelled (Rosenbek et al., 1996; Borders and Brates, 2020). The DD is a four-level score for the swallowing function of patients according to their clinical manifestations; the higher the grade is, the worse the swallowing function of the patients is (Ertekin et al., 2000; Khedr and Abo-Elfetoh, 2010).

### Statistical Analysis

Meta-analysis was performed using Review Manager version 5.3 and heterogeneity was investigated using *I*^2^. If *p* > 0.05 and *I*^2^ ≤ 50%, heterogeneity was not significant and a fixed-effects model was used; otherwise, a random-effects model was used. If heterogeneity existed, we conducted sensitivity analysis and subgroup analysis to find the source of heterogeneity. As for subgroup analysis (frequency), random-effects model was used. A pooled standardized mean difference (SMD) together with 95% confidence interval (CI) were calculated. *p* < 0.05 was regarded as statistically significant.

## Results

### Literature Search Results

**Figure 1:**
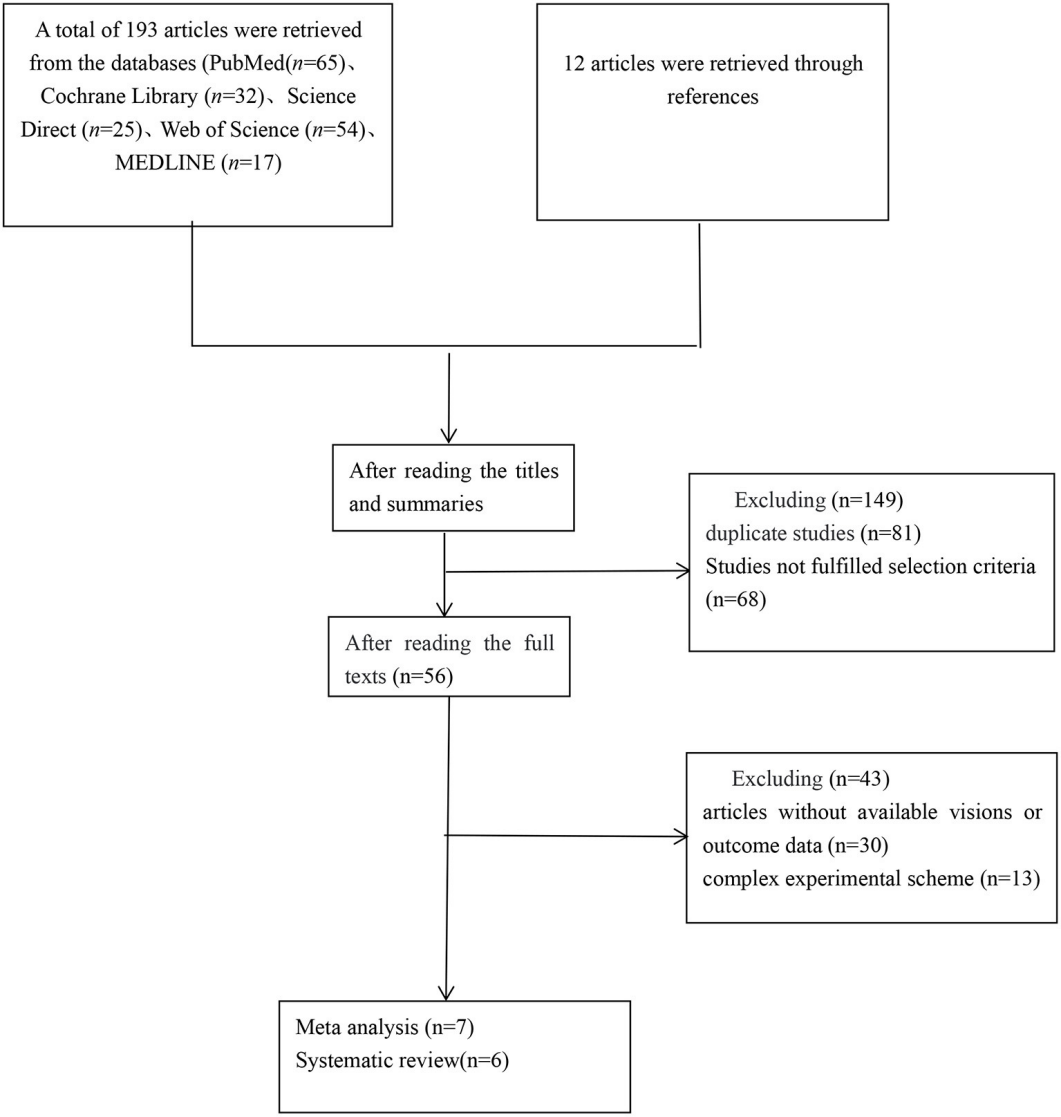
Flow chart of study selection.

A total of 193 articles were retrieved from PubMed (65), Cochrane Library (32), Science Direct (25), MEDLINE (17), and Web of Science (54), and 12 were retrieved through references. After reading titles and abstracts, 81 duplicated studies and 68 irrelevant studies were excluded. After reading the full text, 30 articles without available full-text visions or outcomes data and 13 literature including complex experimental scheme were excluded. Finally, we included seven literature (Khedr et al., 2009; Khedr and Abo-Elfetoh, 2010; Kim et al., 2011; Park et al., 2013, 2017; Lim et al., 2014; Ünlüer et al., 2019), with a total of 186 participants from 14 groups of patients in our meta-analysis, and a qualitative systematic review of six studies (Verin and Leroi, 2009; Cheng et al., 2015, 2017; Du et al., 2016; Tarameshlu et al., 2019; Zhang et al., 2019). The literature screening process and detailed characteristics of included studies are shown in Figure 1 and Table 1.

**Table 1:** Characteristics of the randomized controlled studies.

**References**	***N***		**Age (years)**		**Intervention**		**Time**	**Outcome measures**
	**E**	**C**	**E**	**C**	**E**	**C**		
Kim et al., 2011	10	10	69.8 ± 8.0	68.2 ± 12.6	5 Hz, 100% MT, ipsilesional mylohyoid muscle	Sham stimulation	20 min/day, 5 days/week, 2 weeks	FDS, PAS
Kim et al., 2011	10	10	66.4 ± 12.3	68.2 ± 12.6	1 Hz, 100% MT, contralesional mylohyoid muscle	Sham stimulation	20 min/day, 5 days/week, 2 weeks	FDS, PAS
Khedr et al., 2009	14	12	58.9 ± 11.7	56.2 ± 13.4	3 Hz, 120% MT, ipsilesional esophagus area	Sham stimulation	10 min/day, 5 days	DD
Khedr and Abo-Elfetoh, 2010	6	5	56.7 ± 16	58.0 ± 17.5	3 Hz, 130% MT, both hemisphere esophagus area	Sham stimulation	10 min/day, 5 days	DD
Khedr and Abo-Elfetoh, 2010	5	6	55.4 ± 9.7	60.5 ± 11.0	3 Hz, 130% MT, both hemisphere esophagus area	Sham stimulation	10 min/day, 5 days	DD
Park et al., 2013	9	9	73.7 ± 3.8	68.9 ± 9.3	5 Hz, 90% MT, contralesional pharyngeal	Sham stimulation	10 min/day, 5 days/week, 2 weeks	VDS, PAS
Ünlüer et al., 2019	15	13	67.8 ± 11.9	69.3 ± 12.9	1 Hz, 90% MT, unaffected hemisphere mylohyoid muscle	Conventional therapy	20 min/day, 5 days	PAS
Lim et al., 2014	14	15	59.8 ± 11.8	62.5 ± 8.2	1 Hz, 100% MT, contralesional hemisphere pharyngeal	Conventional therapy	20 min/day, 5 days/week, 2 weeks	FDS, PTT, PAS
Park et al., 2017	11	11	67.5 ± 13.4	69.6 ± 8.6	10 Hz, 90% MT, ipsilesional hemisphere mylohyoid	Sham stimulation	20 min/day, 5 days/week, 2 weeks	DOSS, PAS, VDS
Park et al., 2017	11	11	60.2 ± 13.8	69.6 ± 8.6	10 Hz, 90% MT, bilateral hemisphere mylohyoid	Sham stimulation	20 min/day, 5 days/weeks, 2 weeks	DOSS, PAS, VDS

*T, experimental group; C, control group; Hz, hertz; MT, motor threshold; FDS, functional dysphagia scale; PAS, penetration-aspiration scale; VDS, videofluoroscopic dysphagia scale; DD, dysphagic grade; PTT, pharyngeal transit time; ASHA NOMS, American speech-language hearing association national outcomes measurements system swallowing scale*.

### Quality Assessment of the Included Studies

**Figure 2:**
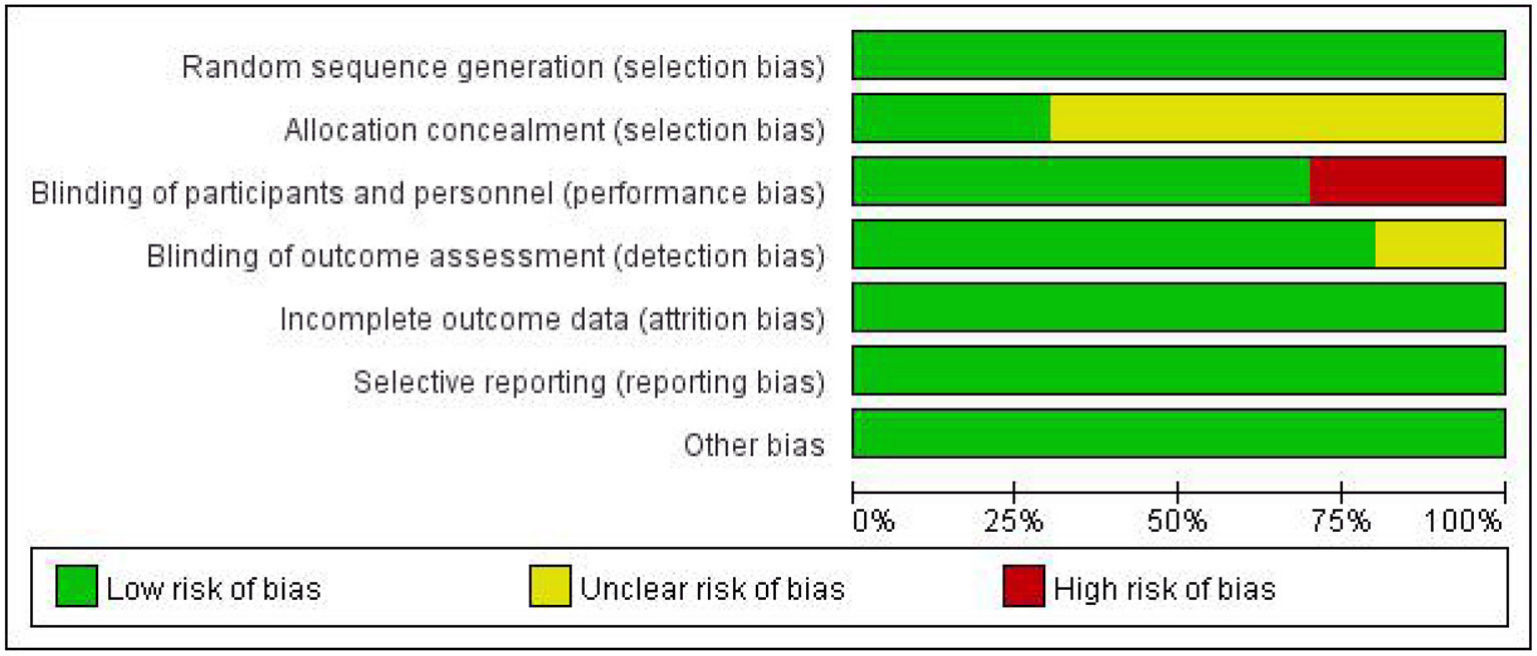
Risk of bias graph.

**Figure 3:**
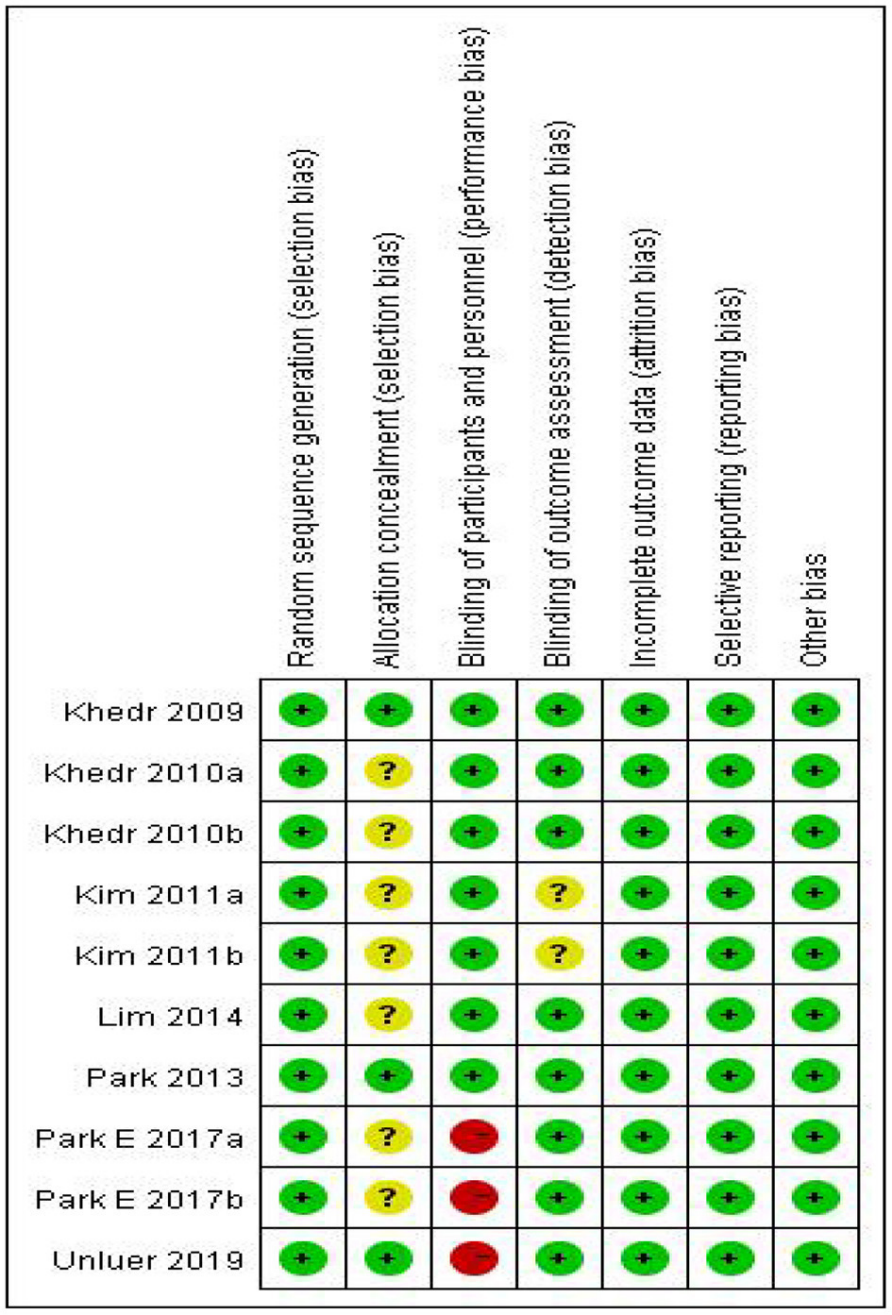
Risk of bias summary.

In our included literature, individual articles designed two experimental groups according to the lesion site, stimulation frequency, and other parameters. According to our audit, it was found that both two experimental groups in the same literature did not interfere with each other. Therefore, we treated each study in these three literatures (Khedr and Abo-Elfetoh, 2010; Kim et al., 2011; Park et al., 2017) as a randomized controlled experiment. So, we got 10 studies from seven articles. Two researchers evaluated the reporting quality of 10 included studies. The integrity of the data was guaranteed to a great extent, but the experimental scheme of Khedr and Abo-Elfetoh (2010), Kim et al. (2011), and Lim et al. (2014) had a risk of selection bias. Researches of Park et al. (2017) and Ünlüer et al. (2019) had an implementation bias (complete blindness of subjects was not achieved). Out of the 10 studies, 2 studies (Khedr et al., 2009; Park et al., 2013) were rated as Grade A, while others were rated as Grade B. The results were presented in Figures 2, 3 and Table 2.

**Table 2:** Methodological quality assessment of the controlled studies.

**References**	**Random sequence generation**	**Allocation concealment**	**Blinding of participants and personnel**	**Blinding of outcome assessment**	**Incomplete outcome data**	**Selective reporting**	**Other bias**	**Grade**
Khedr et al., 2009	Low risk	Low risk	Unclear	Low risk	Low risk	Low risk	Low risk	A
Khedr and Abo-Elfetoh, 2010	Low risk	Unclear	Low risk	Low risk	Low risk	Low risk	Low risk	B
Khedr and Abo-Elfetoh, 2010	Low risk	Unclear	Low risk	Low risk	Low risk	Low risk	Low risk	B
Kim et al., 2011	Low risk	Unclear	Low risk	Unclear	Low risk	Low risk	Low risk	B
Kim et al., 2011	Low risk	Unclear	Low risk	Unclear	Low risk	Low risk	Low risk	B
Lim et al., 2014	Low risk	Unclear	Low risk	Low risk	Low risk	Low risk	Low risk	B
Park et al., 2013	Low risk	Low risk	Low risk	Low risk	Low risk	Low risk	Low risk	A
Park et al., 2017	Low risk	Unclear	High risk	Low risk	Low risk	Low risk	Low risk	B
Park et al., 2017	Low risk	Unclear	High risk	Low risk	Low risk	Low risk	Low risk	B
Ünlüer et al., 2019	Low risk	Low risk	High risk	Low risk	Low risk	Low risk	Low risk	B

Additionally, the outcome indicators in recruited articles were different; we have to use a random-effects model and SMD to reduce the inevitable heterogeneity. In future experiments and studies, we will try to select the same standard for unified data analysis of randomized controlled trials.

### Results of Systematic Review

A total of six studies with a small sample size or non-randomized controlled trial studies (Verin and Leroi, 2009; Cheng et al., 2015, 2017; Du et al., 2016; Tarameshlu et al., 2019; Zhang et al., 2019) were included in the systematic review. They investigated the efficacy of rTMS on patients with dysphagia after stroke from different perspectives. Tarameshlu et al. (2019), Zhang et al. (2019), Du et al. (2016), and Verin and Leroi (2009) all supported that rTMS treatment over the tongue area of the motor cortex may facilitate the recovery of dysphagia after stroke. However, not all studies proved the efficacy of rTMS for the treatment of dysphagia. The research group of Cheng et al. published relevant studies of rTMS on swallowing disorders in chronic post-stroke patients in 2015 and 2017, respectively (Cheng et al., 2015, 2017). In the previous study, they pointed out that 5-Hz rTMS might improve swallowing flexibility and promote the recovery of swallowing function *via* improving tongue muscle function, but the latter study showed that the efficacy retention time of rTMS was relatively short, and the long-term therapeutic effect could not be observed. The two studies were conducted by the same authors and design, but the result was different, which probably resulted from the small sample size. It was found that the theoretical basis of these studies was based on the hypothesis of transcallosal imbalance for bilateral hemisphere, which also suggested that functional recovery of dysphagia after stroke critically depends on neural plasticity.

### Results of Meta-Analysis

#### Effects on Deglutition Disorders

**Figure 4:**
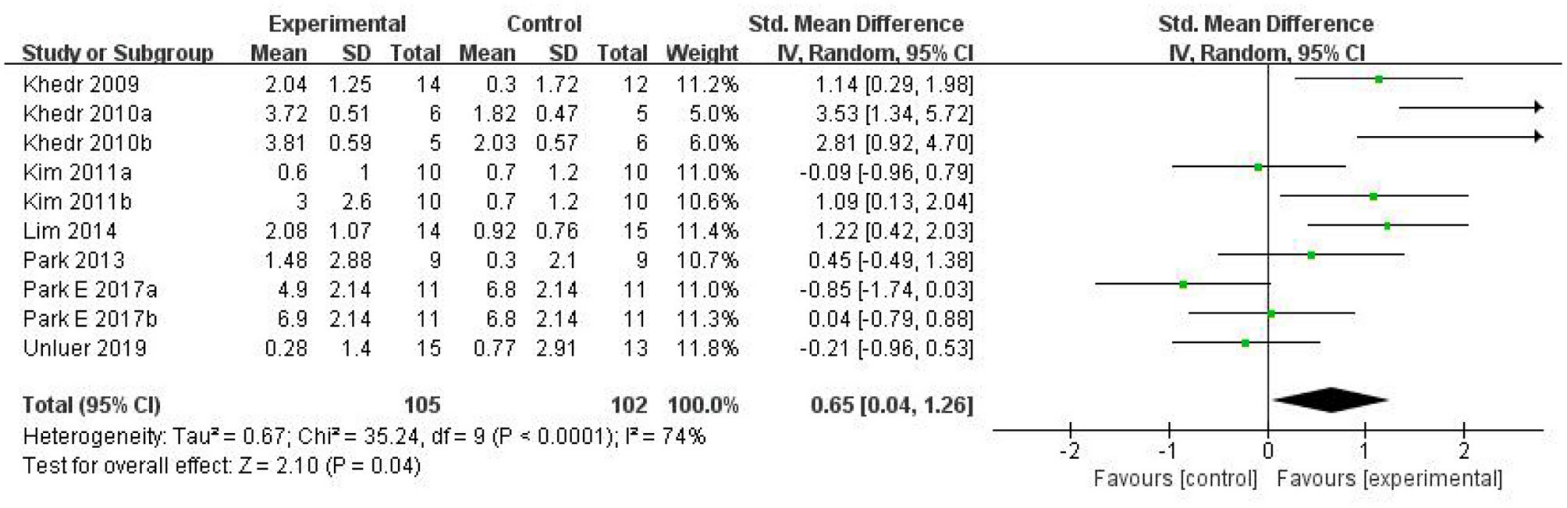
The effects of rTMS on deglutition disorders.

Ultimately, 10 studies involving 186 subjects were included in the meta-analysis. The pooled results indicated that rTMS had a significant effect on the improvement of swallowing function after stroke compared with the control group [SMD = 0.65, 95% CI (0.04, 1.26), *p* = 0.04, fixed-effects model]. However, there was great heterogeneity among studies (*p* = 0.0004, *I*^2^ = 74%). Due to the small number of included control groups, funnel plot analysis was deemed not useful and hence not conducted. We attempted to conduct sensitivity analysis to find out the main sources of heterogeneity, but none of the included studies could explain the main causes of heterogeneity after removing each study one by one. Therefore, we can only reduce the heterogeneity statistically using a random-effects model. The results showed that rTMS may significantly improve swallowing function [SMD = 0.65, 95% CI (0.04, 1.26), *p* = 0.04, random-effects model]. The results are presented in Figure 4.

#### Subgroup Analysis of the Effects of rTMS for Swallowing Function (Frequency)

**Figure 5:**
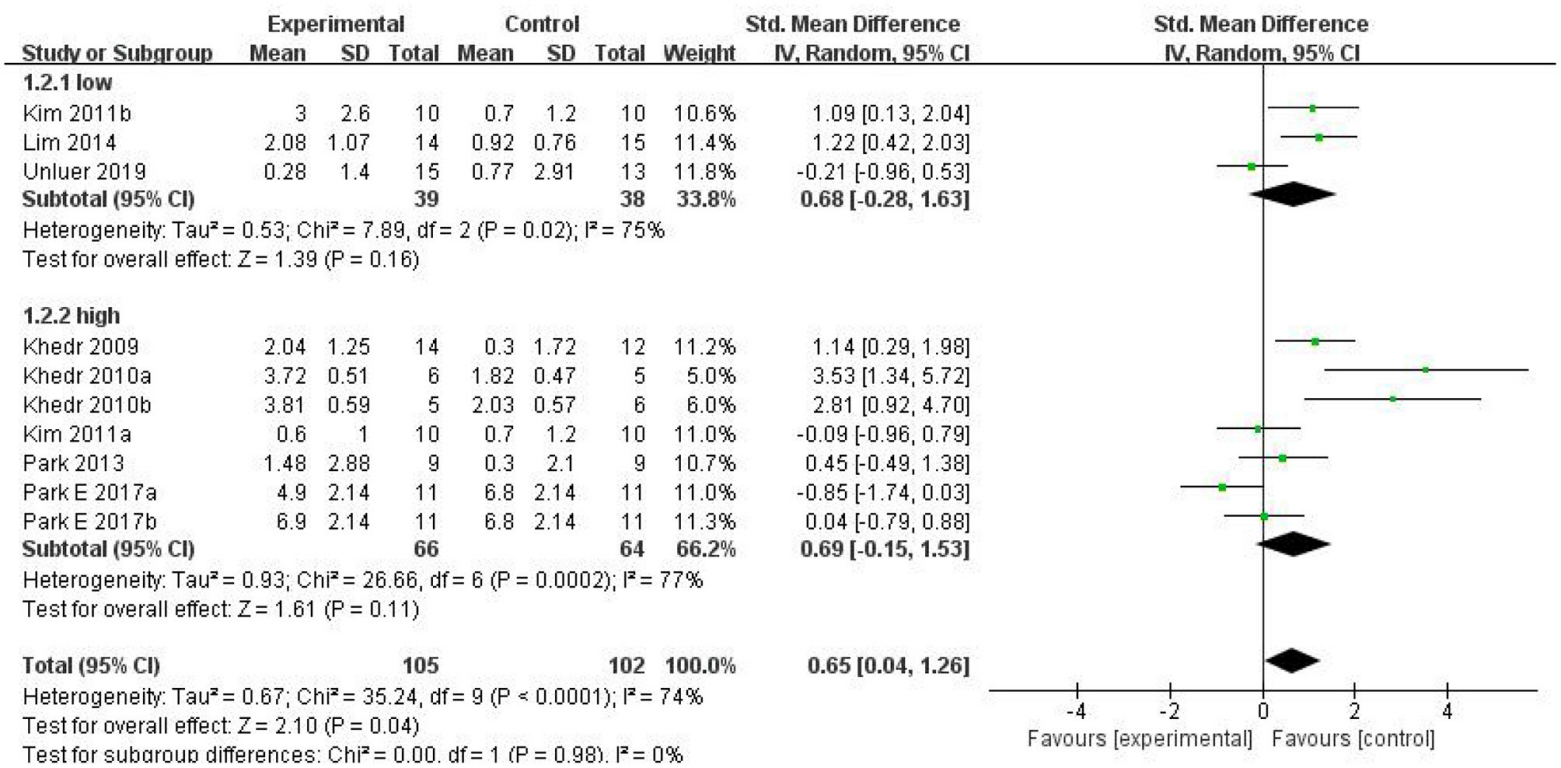
Subgroup analysis of the effects of rTMS for swallowing function (frequency).

In clinical practice, rTMS mainly acts on the brain in two modes of stimulation: LF-rTMS and HF-rTMS. Low-frequency cortical stimulation can inhibit cortical excitability and might play a role in long-term inhibition. However, high-frequency stimulation can increase the excitability of the site of stimulation and achieve long-term effect. In our study, subgroup analysis was conducted according to different frequencies. The combined results showed that rTMS was superior to conventional rehabilitation training in treating dysphagia after stroke. However, both the low-frequency stimulation group [SMD = 0.68, 95% CI (−0.28, 1.63), *p* = 0.16] and high-frequency stimulation group [SMD = 0.69, 95% CI (−0.15, 1.53), *p* = 0.11] had the same efficacy as conventional rehabilitation for dysphagia after stroke. The differences between the two subgroups were not statistically significant, which may stem from the fact that the number of studied patients was too small. Although the combined result generated positive outcomes, the dominant effect was not significant. Specific results are shown in Figure 5.

### Adverse Events

In our included studies, four studies reported that no adverse effects occurred after rTMS treatment, and eight studies showed no adverse effects during or after rTMS treatment. Only two studies (Lim et al., 2014; Ünlüer et al., 2019) reported mild noise sensation, headache, and dizziness during treatment, but the above sensations would disappear spontaneously shortly after treatment without further interventions. Although it has been reported that high-frequency stimulation may cause the occurrence of epilepsy, none of the participants in the included studies experienced epilepsy. However, for safety concerns, therapeutic protocols of rTMS to treat dysphagia should be developed to avoid various side effects.

## Discussion

Our study suggested that rTMS treatment may improve the swallowing function of patients with dysphagia after stroke, but the efficacy of either the LF-rTMS or HF-rTMS group was not significantly different from that of the conventional treatment group. This observation differed from what was reported by Liao et al. (2017). The possible reasons were speculated as follows. First of all, the number of included participants was small in our analysis, and the combination of LF-rTMS and HF-rTMS was with a slight advantage over conventional treatment for treating dysphagia. Thus, further subgroup analysis led to a smaller number of included patients and had an overall high risk of bias. Secondly, after careful observation of the studies by Khedr and Abo-Elfetoh (2010), and Khedr et al. (2009), it was found that the patients selected by their research group all had dysphagia caused by brain stem or medulla oblongata lesions, while other studies were dominated by cortical lesions. This leads us to speculate that rTMS has different results for different lesion sites, especially cortical and subcortical lesions. In a word, combined analysis confirmed the advantage of rTMS, which was consistent with the results of Yang et al. (2015) and Pisegna et al. (2016). Distinct from the analyses of Yang et al. (2015) and Pisegna et al. (2016) we evaluated the effect of rTMS as the only intervention methods for patients without using other non-invasive central stimulation techniques (rTMS and tDCS) concurrently to avoid the interference.

Review of the included studies found that most of the studies were based on the interhemispheric inhibition theory and investigated two kinds of treatment methods—LF-rTMS and HF-rTMS. LF-rTMS is supposed to suppress the cortical excitability of healthy hemisphere while HF-rTMS activates excitability of stroked hemisphere. The combination of two methods helps improve bilateral imbalance of the brain so as to improve patients' swallowing functions. Interhemispheric inhibition theory is now commonly used in clinic. However, a few studies (Park et al., 2013, 2017) used the hypothesis of compensatory model to explain the effect of high-frequency stimulation on healthy hemispheres for the improvement of deglutition. Hamdy et al. (1996, 1998) used TMS to study the mechanisms of post-stroke deglutition disorders and found that the swallowing muscle group represents asymmetrical cortical functional areas in bilateral hemispheres. Swallowing function requires the common input information from bilateral cortices. This means that the recovery of post-stroke deglutition may probably depend on functional compensation of the healthy hemisphere. Therefore, the compensation model is also a strong argument for the therapeutic effect of rTMS on swallowing function recovery. In recent years, researchers have further developed the “bimodal balance recovery model” (Di Pino et al., 2014; Sankarasubramanian et al., 2017), arguing that the previous two models are no longer opposite, but are integrated with each other to achieve brain neuroplasticity. So, maybe rTMS stimulated the plasticity involved in swallowing control by acting on different circuits (Cantone et al., 2021). The above assumptions attempt to explain the complex mechanisms of rTMS on dysphagia. The review of Cantone et al. has shown that rTMS could modify cortical excitability, plasticity, and connectivity interacting in the pathophysiology of the impairment (Cantone et al., 2020). Additionally, restoration of maladaptive plasticity is another mechanism for the effect of rTMS (Hulme et al., 2013; Cantone et al., 2018, 2020; Vinciguerra et al., 2020). All these can promote the plasticity of neurons in the brain and achieve functional recovery.

However, there are still certain limitations with our analyses. First of all, the main limitations of the review are the small number of studies and participants included in our review. Secondly, only subgroup analysis for frequency was performed in our paper, whereas heterogeneity was observed in both subgroups. The efficacy of rTMS could also be affected by other parameters such as stimulation sites and locations of lesions. Thirdly, we chose PAS and DD as evaluation criteria, but did not include other evaluation criteria, which also resulted in a certain bias in the inclusion process of the article. In order to better serve the clinical practice, we will sort out all commonly used clinical evaluation criteria and collect relevant literature for data analysis in the later stage. Further clinical controlled trials should be conducted to explore the influence factors on the efficacy and mechanism of rTMS in treating post-stroke dysphagia.

## Conclusion

We conducted a systematic review and meta-analysis of rTMS for dysphagia after stroke, and the results showed that rTMS is more effective than conventional training for the recovery of dysphagia. However, subgroup analysis of HF-rTMS and LF-rTMS did not show significant efficacy for post-stroke dysphagia when compared with conventional rehabilitation, which was probably due to a small number of included studies. Thus, more large trials were needed for further study. Our study also found that both interhemispheric inhibition theory and compensatory model may play a role in the therapeutic effect of rTMS in treating dysphagia. More clinical studies are needed to help develop better treatment plans for these patients.

## Data Availability Statement

The original contributions presented in the study are included in the article, further inquiries can be directed to the corresponding author/s.

## Author Contributions

XC, XZ, XW, and XL assisted in literature review, quality assessment, and data analysis for our review. All authors contributed to the article and approved the submitted version.

## Funding

The research was supported by the 2020 District Level Scientific Research Project of Longhua District Medical (ID:2020040) and Health Institutions and Shenzhen Longhua District Rehabilitation Medical Equipment Development and Transformation Joint Key Laboratory.

## Conflict of Interest

The authors declare that the research was conducted in the absence of any commercial or financial relationships that could be construed as a potential conflict of interest.

## Publisher's Note

All claims expressed in this article are solely those of the authors and do not necessarily represent those of their affiliated organizations, or those of the publisher, the editors and the reviewers. Any product that may be evaluated in this article, or claim that may be made by its manufacturer, is not guaranteed or endorsed by the publisher.
